# Potential of Induced Pluripotent Stem Cells (iPSCs) for Treating Age-Related Macular Degeneration (AMD)

**DOI:** 10.3390/cells5040044

**Published:** 2016-12-08

**Authors:** Mark Fields, Hui Cai, Jie Gong, Lucian Del Priore

**Affiliations:** Department of Ophthalmology and Visual Science, Yale School of Medicine, Yale University, 300 George St., Suite 8100, New Haven, CT 06511, USA; huey.cai@yale.edu (H.C.); jie.gong@yale.edu (J.G.); lucian.delpriore@yale.edu (L.D.P.)

**Keywords:** age-related macular degeneration, induced pluripotent stem cell, retinal pigment epithelium, Bruch’s membrane, disease modeling

## Abstract

The field of stem cell biology has rapidly evolved in the last few decades. In the area of regenerative medicine, clinical applications using stem cells hold the potential to be a powerful tool in the treatment of a wide variety of diseases, in particular, disorders of the eye. Embryonic stem cells (ESCs) and induced pluripotent stem cells (iPSCs) are promising technologies that can potentially provide an unlimited source of cells for cell replacement therapy in the treatment of retinal degenerative disorders such as age-related macular degeneration (AMD), Stargardt disease, and other disorders. ESCs and iPSCs have been used to generate retinal pigment epithelium (RPE) cells and their functional behavior has been tested in vitro and in vivo in animal models. Additionally, iPSC-derived RPE cells provide an autologous source of cells for therapeutic use, as well as allow for novel approaches in disease modeling and drug development platforms. Clinical trials are currently testing the safety and efficacy of these cells in patients with AMD. In this review, the current status of iPSC disease modeling of AMD is discussed, as well as the challenges and potential of this technology as a viable option for cell replacement therapy in retinal degeneration.

## 1. Introduction

Progress in the area of regenerative medicine has begun to unlock new opportunities in the way health care providers approach treatment of debilitating disorders. Efforts to develop viable treatments, such as cell replacement therapy, have begun to become a reality for such disorders as diabetes, Parkinson, Alzheimer, and multiple sclerosis [1,2]. The field of ophthalmology is no different and has seen dramatic advancements in the area of stem cell-based treatments. Therapies are being developed for such visual impairment disorders as age-related macular degeneration (AMD), a leading cause of blindness in the United States and Western Europe, which has a significant impact on the quality of life of affected individuals [3]. Pertinently, a search of www.ClinicalTrials.gov reveals a number of stem cell-based therapies for the treatment of AMD that have progressed to human clinical trials. The main objective of these trials is to test the safety and efficacy of these treatments in patients with AMD [4,5].

The development of induced pluripotent stem cell (iPSC) technology has presented a paradigm shift in the field of stem cell biology and provides an alternative source of pluripotent cells. The pioneering accomplishment in 2006 by the Yamanaka group had very important implications for the field with the novel discovery that differentiated somatic cells can be induced into a pluripotent state using a cocktail of proteins called “Yamanaka factors” [6]. These “reprogrammed” cells, therefore have the ability to be differentiated into any cell type in the body. With this advancement has come many concerns, such as ethical use and a continuous source of embryonic stem cells that have hindered clinical development. With reprogrammed cells these concerns are negated and the possibility of developing patient-specific therapies using autologous cells has been introduced.

Induced pluripotent stem cell technology provides a patient-specific source of cells that, from a clinical standpoint, affords a potential cell replacement therapy that may circumvent immune rejection [7,8]. The fact that these cells can be directly generated from the patient affords investigators the opportunity to model a specific disease and provides a relevant investigational tool that is an alternative to traditional animal models [9,10,11]. Use of iPSC-derived cells for disease modeling can allow for the understanding of the pathology and cell biology of retinal diseases such as AMD, and help elucidate the morphological changes attributed to the aging process and progression of disease [12,13]. These models can also lead to the development of platforms for drug screening and safety studies. Given the utility of iPSCs, both as a research tool to understand disease pathophysiology and as a therapeutic for cell replacement therapy, their potential continues to be investigated.

We present a review herein of the current state of iPSCs for the treatment of such retinal degenerative diseases as AMD. In particular, the merit of iPSC-derived disease models to understand the pathophysiology of geographic atrophy (GA), as well as the status of ongoing clinical trials using embryonic stem cells (ESCs) or iPSCs as cell sources will be discussed.

## 2. AMD and Bruch’s Membrane Pathology

Age-related macular degeneration is a multifactorial disease that affects the outer retina, choriocapillaris, retinal pigment epithelium (RPE), and Bruch’s membrane (BM) [14,15]. The disease is characterized by structural changes within BM which then leads to cellular changes in the RPE including loss of RPE cells and the eventual development of advanced forms of the disorder, such as GA.

Traditionally, AMD has been classified into two types, exudative (neovascular or “wet”) AMD or atrophic (“dry”) AMD [3,15,16,17]. One of the earliest clinical manifestations of AMD is the focal deposition of acellular, polymorphous debris between the RPE and BM called drusen [15]. With age, drusen can accumulate and eventually cause damage to the native RPE cells disrupting crucial cellular functions such as maintaining the integrity of the retina and choriocapillaris, including phagocytosis of the distal tips of photoreceptor outer segments, transport and isomerization of bleached visual pigments, maintenance of the blood-outer retinal barrier and maintaining perfusion of the subjacent choriocapillaris [18,19,20,21,22,23,24,25,26,27,28,29,30,31].

In atrophic or “dry” AMD, there is a progressive loss of the RPE and subsequent loss of photoreceptors and/or choriocapillaris. The decline of this tissue inevitably leads to loss of vision, observed clinically as central and paracentral scotomas [15,32]. In exudative or “wet” AMD, abnormal expression of angiogenic factors such as vascular endothelial growth factor (VEGF) can cause neovascularization to arise from the neural retina or choriocapillaris within BM eventually finding its way into the subretinal space and/or subretinal pigment epithelium [15,32]. In many cases, the exudative form can progress and lead to severe vision loss, but with the introduction of intravitreal antiangiogenic therapy a new standard of care has become a very effective treatment to slow or reverse vision loss in many individuals [15,33].

The contribution of BM alterations to AMD pathogenesis is significant, particularly in the context of the development of a cell replacement therapy for advanced disease. These alterations include diffuse BM thickening; accumulation of drusen, basal laminar, and basal linear deposits [34,35]; collagen cross-linking in the inner and outer collagen layer; calcification and fragmentation of the elastin layer [36]; and BM lipidization [36,37,38,39]. It has also been reported that structural changes within BM precede cellular changes in the RPE by one or two decades [34,40].

Importantly, these age-related changes within BM can potentially have a negative effect on the function of transplanted cells [34,35,36,37,38,40,41,42,43,44,45,46,47]. It has been demonstrated that disease and/or damage within human-aged BM is an important factor that adversely affects transplant survival and proliferation. For example, aging of human BM, or damage induced by surgical manipulation of choroidal neovascularization, can reduce the ability of transplanted RPE cells to attach to human BM, survive after transplantation, and proliferate to repopulate this structure [42,45,48,49]. Aging of human BM also reduces the ability of human RPE cells seeded on this surface to ingest rod outer segments [50]. Thus, it is clear that in the advanced stages of AMD, namely GA, age-related changes to BM can adversely affect the successful transplantation of cells. Interestingly, we have previously shown that non-enzymatic nitration of the basement membrane is a relevant model system to study BM pathology and can affect RPE dysfunction, such as altered VEGF secretion, phagocytic ability, and expression of complement regulatory proteins in a manner that mimics the effects of BM aging in AMD [50,51,52,53]. We have further shown that cleaning and coating the surface of BM or nitrite-modified extracellular matrix (ECM) with such proteins as laminin, fibronectin, and vitronectin can reverse the effects of damage associated with an aged and/or diseased BM [52,54]. Current trials have transplanted stem cell-derived RPE as cell suspensions but the pathology of BM may give credence to the use of a scaffold as a substrate that would allow RPE to attach and proliferate as polarized monolayers [55]. These scaffolds can be made of materials that include collagen and poly(lactic-co-glycolic acid) (PLGA) and it has been demonstrated that a basal support membrane is critical to long-term RPE survival after implantation [56]. It will be of interest to determine the most efficient transplantation method as both single cell suspensions and membrane supports show efficacy [56,57].

## 3. Induced Pluripotent Stem Cells

Induced pluripotent stem cell (iPSC) technology was originally developed by Yamanaka and colleagues in 2006 [6]. The group demonstrated that the combination of transcriptional regulators SRY (sex determining region Y)-box 2 (SOX2), octamer-binding transcription factor 3/4 (OCT3/4), kruppel-like factor 4 (Klf4), and Myc (c-MYC) had the ability to reprogram mouse fibroblasts into a pluripotent stem-like state called iPSCs. The combination of these proteins has been coined the “Yamanaka factors” after its inventor. Methods to generate iPSCs have evolved rapidly since the introduction of the technology in 2006 and have been used to reprogram somatic cells from a number of species, including the rat [58], dog [59], rhesus monkey [60], and human [61,62].

A hallmark of these cells is their ability to differentiate into three germ layer cell types (mesoderm, ectoderm, and endoderm) verifying pluripotency [6,61,63,64]. Given their pluripotent attributes, these cells can potentially become any cell type in the body, making them a valuable resource in the area of regenerative medicine and disease modeling.

## 4. Induced Pluripotent Stem Cell-Derived Retinal Pigment Epithelium

In advanced atrophic AMD or GA there is a loss of RPE, photoreceptors, and possibly the choriocapillaris in affected areas, which will require the introduction of replacement cells and/or trophic factors to reconstruct the outer retinal anatomy [32]. Loss of vision is typically due to atrophy of the RPE and photoreceptors with secondary loss of the choriocapillaris. The RPE is important for photoreceptor survival and function, and loss of this cell type is involved in the pathophysiology of atrophy in AMD [65]. The rationale for transplantation of RPE cells is clear and the potential for cell-based therapy has been investigated in both animals and humans [66,67,68].

**Figure 1:**
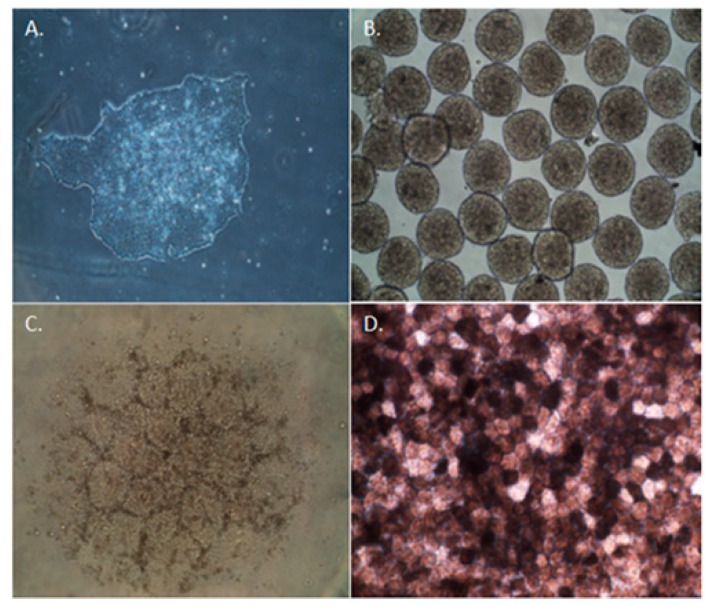
Differentiation of induced pluripotent stem cells (iPSCs) toward a retinal pigment epithelium (RPE) fate. Undifferentiated iPSC colony at day 0 (**A**); embryonic bodies form by day 7 (**B**); and eventual formation of neural aggregates (**C**) by day 14; A pigmented monolayer of iPSC-derived RPE cells forms by day 45 of the differentiation process (**D**). With the full permission of all authors of the original publication, Figure 3 of [76] has been included here.

**Figure 2:**
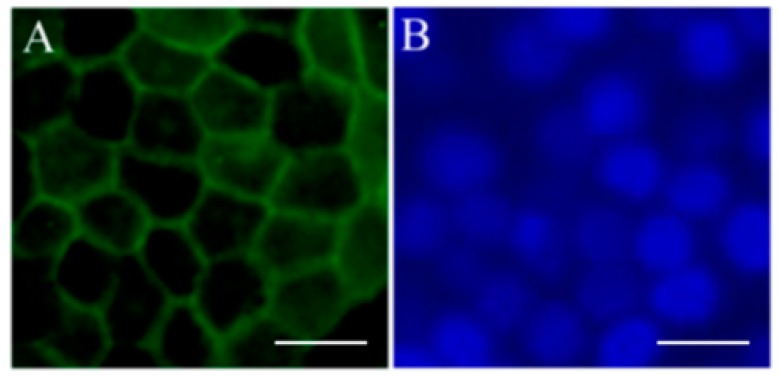
Expression of retinal pigment epithelium (RPE) cell markers in induced pluripotent stem cells (iPSC)-derived RPE. Immunofluorescent staining of RPE marker ZO-1 (**A**) in pigmented iPSC-derived RPE cells; (**B**) DAPI image of same cells. Scale bars = 50 μm. With the full permission of all authors of the original publication, Figure 4 of [76] has been included here.

**Figure 3:**
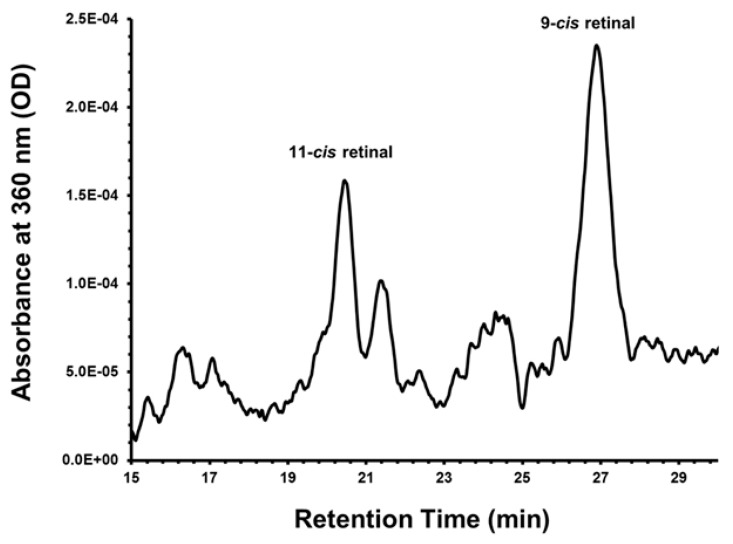
Retinoid metabolism in induced pluripotent stem cell (iPSC)-derived retinal pigment epithelium (RPE) monolayers. iPSC-RPE cells were cultured in 6-well plates until a confluent and pigmented monolayer was observed. Retinoid profiles were taken by monitoring the HPLC at 360 nm, two days post administration of 5 μmol/L all-*trans* retinol (atRol) in 1% bovine serum albumin. iPSC-RPE cell cultures express 11-*cis*-retinal indicating functional retinoid metabolism. No hydroxylamine was used in these experiments; therefore, oximes are not detected. Note that significant quantities of the more stable 9-*cis* isomer are also formed with the administration of all-*trans* retinol. With the full permission of all authors of the original publication, Figure 6 of [76] has been included here.

The use of iPSC-derived RPE cells may provide an unlimited source of cells, an inherent limitation when using other potential sources such as donor adult or fetal RPE cells. Use of patient-specific iPSC-derived RPE cells offers an autologous source of cells that are suitable as a research tool to understand disease mechanisms. Many groups, including ours, have differentiated iPSCs into RPE cells successfully and established reproducible protocols [69,70,71,72,73,74,75]. Our laboratory has demonstrated the ability to differentiate a human iPSC line of RPE cells using an established protocol (Figure 1) [69,76]. These cells are morphologically similar to native RPE cells and express RPE-specific markers, such as zonula occludens protein-1 (ZO-1) (Figure 2). These cells also have the potential to perform critical functions such as the ability to process retinoids similar to native RPE. (Figure 3) [76]. The in vivo function and safety of iPSC-derived RPE cells have also been demonstrated in animal models of retinal degeneration [71,77,78].

## 5. Use of iPSC-Derived RPE to Model Age-Related Macular Degeneration

As mentioned, one of the advantages of using iPSCs is the ability to model a specific disease in vitro by developing a disease phenotype and intervening through drug screening [79]. Ocular diseases, such as glaucoma and Best disease, have been modeled using iPSCs. These models produced disease phenotypes that have advanced our understanding of the genetics of disease [80,81]. For example, Singh et al. demonstrated defective photoreceptor outer segment degradation and disposal as well as reduced fluid transport in iPSC-derived RPE derived from patients with the RPE-specific protein bestrophin-1 (BEST1) mutation [80]. Disease modeling with iPSC derived from monogenic degenerative disorder will benefit greatly from this technology [9,12].

It has been difficult, historically, to model age-related disorders, such as GA, in the animal, particularly in the lower vertebrates such as the mouse who do not have a macula [82]. While animal models are an extremely valuable and indispensable tool for research, developing models of GA using human iPSCs from patients with AMD that could mimic or accelerate the aging process could prove valuable. Moreover, iPSC phenotypes from patients with a particular disease, such as exudative or atrophic AMD, may differ from what is observed in the animal and serve as a valuable source for comparative study [82,83].

Several studies have demonstrated that risk factors such as advanced age, race, and mutations in complement alleles such as complement factor H are associated with AMD [84]. It is clear that the deleterious effects of drusen accumulation on BM contribute to RPE dysfunction and chronic inflammation [51], which are both hallmarks of AMD pathology. Model systems that mimic the effects of BM aging can be used to determine the contribution of ECM damage on the cellular function and pathology of the overlying RPE cells [51,52,85]. Moreover, the use of patient-specific iPSC-derived RPE cells from patients with high and low risk alleles for AMD may reveal how these alterations contribute to RPE dysfunction and atrophy. This area is particularly valid in light of the disorder being an interplay between multiple genetic susceptibility factors and environmental components [86]. Continued advancement in this area will lead to a novel understanding of a multifactorial and complex disease.

## 6. Current Status of iPSC Therapies for the Treatment of Retinal Disorders

The use of iPSCs as an option for cell replacement therapy in humans is the ultimate end-goal of this technology. There are a number of advantages to using iPSCs including alleviation of ethical concerns that have hampered ESC clinical development. Moreover, iPSCs present the opportunity to produce autologous cells and, thus avoid the need to find a human leukocyte antigen (HLA)-compatible cell donor and the need for immunosuppression [87]. Table 1 describes the interventional trials that are currently (2016) cited at the www.ClinicalTrials.gov registry and are now in progress investigating the safety and efficacy of human ESC-derived RPE for the treatment of disorders, such as atrophic AMD and Stargardt macular dystrophy [65,88,89]. There are also a number of trials being conducted internationally investigating the safety and efficacy of human ESC-derived RPE in the treatment of exudative and atrophic AMD. As of 2016, leading the way in ongoing trials labeled as “interventional” are such companies as the Astellas Institute for Regenerative Medicine and Pfizer. Groups at The Federal University of São Paulo, the Southwest Hospital (China), Regenerative Patch Technologies, LLC, and Cell Cure Neurosciences Ltd. are sponsoring “interventional” trials that are actively recruiting. Interestingly, the Regenerative Patch Technologies, LLC trial is investigating the use ESC-derived RPE seeded on a polymeric substrate (Table 1). Long-term survival of these cells on these types of substrates will be of great interest in determining the most efficient and efficacious means of transplantation. It should be noted that there are groups investigating the use of other sources of stem cells such as those derived from the human brain and grown as neurospheres (human central nervous system stem cells; HuCNS-SC^®^) (Table 2) [90]. Stem Cells, Inc. recently completed a trial that tested the safety and efficacy of HuCNS-SC in the treatment of AMD [89].

Current efforts to conduct clinical trials using iPSC-derived RPE have been extremely limited. To date, there has been one trial attempt to treat exudative AMD using autologous iPSC-derived RPE cells [65,88,89,91,92]. In 2014, a Japanese woman with exudative macular degeneration was implanted with an iPSC-derived RPE sheet generated from her own fibroblasts [93]. Even though the first patient suffered “no serious adverse effects” of treatment, the trial has been put on hold due to the discovery of genetic mutations identified in the iPSCs that were to be used in a second patient in 2015 [94,95]. While it is not clear whether these genetic abnormalities were induced during the reprogramming process or originated from the patient’s somatic cells, these genomic instabilities must be evaluated before entering human trials. Moving forward, one goal of Sugita et al., is to use iPSCs from partially-matched donors rather than the autologous cells from the same individual to avoid the potential of genomic abnormalities. Sugita and colleagues have demonstrated that cells from major histocompatibility complex (MHC) homozygous donors can be used in histocompatible recipients for treatment of retinal disease [96]. In that study, investigators transplanted iPSC-derived RPE cells in MHC homozygous animals and found no immune response or rejection of iPSC-derived RPE allografts when using MHC-matched animal models without immunosuppression. These studies show that if the donor is MHC or HLA-matched, iPSC-derived RPE cell donor transplantation may be successful with little to no immunosuppression [96,97]. These promising results for iPSC-derived cell types are continuing to be investigated. It has recently been reported that the Riken Center for Developmental Biology will resume its clinical trial using donor cells [98].

While there is much promise in moving iPSC technology to clinical applications, there is still work to be done in our understanding of tumorigenicity and cell survival post-transplantation before entering the human. Work done by Kanemura et al., has demonstrated in animal models that the tumorigenic potential of iPSC-derived RPE is negligible in rodent models. More in vivo studies will need to be conducted [99]. The initial iPSC lines will have to be extensively characterized, particularly in light of the U.S. Food and Drug Administration (FDA) and regulatory approval to ensure safety. Given that the FDA classifies pluripotent stem cells as human cellular and tissue products that are “more than minimally manipulated and used in a non-homologous manner”, it is critical that the issues as discussed above be addressed before iPSC therapies progress to Phase I–III clinical trials [100]. Table 3 describes examples of “observational” trials investigating the feasibility for use of iPSC-derived RPE cells for the treatment of various types of macular degeneration. In addition to the above concerns, the issue of cost must be addressed given the extensive amount of time and resources to develop lines and then test safety [94]. As the technology develops this cost may be greatly reduced.

## 7. Conclusions

The use of induced pluripotent stem cells for the treatment of age-related macular degeneration holds great potential, but there are still important obstacles that must be addressed. iPSC technology has afforded novel understanding in the area of retinal degeneration through autologous iPSC development and disease modeling. Moving forward, it will be important to optimize reprogramming methods, develop efficient methods to produce large numbers of cells ready for clinical use, test safety and integrity, and understand the long-term survival profiles of cells post-transplantation. Even with these current limitations, the 2006 discovery has the unique opportunity to make new inroads in regenerative medicine and change the face of the field.

## Figures and Tables

**Table 1:** ESC and iPSC-derived RPE-based cell types in clinical trials for inherited and non-inherited macular degeneration. Study type: Interventional. Last updated 25 October 2016.

Sponsor	Cell Type or Intervention	Condition	Phase of Trial	Type of Delivery (Intervention)	ClinicalTrials.gov Identifier	Status
Regenerative Patch Technologies, LLC	CPCB-RPE1; human ESC-derived RPE seeded on polymeric substrate	Advanced, dry age-related macular degeneration (AMD)	Phase I and II	Subretinal implantation	NCT02590692	Recruiting
Astellas Institute for Regenerative Medicine	MA09-hRPE; human ESC-derived RPE	Advanced, dry age-related macular degeneration (AMD)	Phase I and II	Subretinal implantation	NCT01344993	Completed
Astellas Institute for Regenerative Medicine	MA09-hRPE; human ESC-derived RPE	Stargardt macular dystrophy (SMD)	Phase I and II	Subretinal implantation	NCT01469832	Completed
CHABiotech Co., Ltd.	MA09-hRPE; human ESC-derived RPE	Advanced, dry age-related macular degeneration (AMD)	Phase I and II	Subretinal implantation	NCT01674829	Unknown
Astellas Institute for Regenerative Medicine	MA09-hRPE; human ESC-derived RPE	Stargardt macular dystrophy (SMD)	Phase I and II	Subretinal implantation	NCT01345006	Completed
University of California, Los Angeles	MA09-hRPE; human ESC-derived RPE	Myopic macular degeneration (MMD)	Phase I and II	Subretinal implantation	NCT02122159	Withdrawn
Cell Cure Neurosciences, Ltd.	OpRegen: human ESC-derived RPE	Advanced, dry-form age-related macular degeneration (geographic atrophy, GA)	Phase I and II	Subretinal implantation	NCT02286089	Recruiting
CHABiotech Co., Ltd.	MA09-hRPE; human ESC-derived RPE	Stargardt macular dystrophy (SMD)	Phase I	Subretinal implantation	NCT01625559	Unknown
Federal University of São Paulo	Human ESC-derived RPE in suspension; human ESC-derived RPE seeded in a substrate	Age-related macular degeneration	Phase I and II	Subretinal implantation	NCT02903576	Recruiting
		Exudative, age-related macular degeneration				
Pfizer	PF-05206388; human ESC-derived RPE	Acute, wet age-related macular degeneration	Phase I	Intraocular implantation	NCT01691261	Active, not recruiting
		Rapid vision decline				
Southwest Hospital, China	Human ESC-derived RPE	Macular degeneration, Stargardt macular dystrophy	Phase I	Subretinal transplantation	NCT02749734	Recruiting

ESC, embryonic stem cell; iPSC, induced pluripotent stem cell; RPE, retinal pigment epithelium.

**Table 2:** Stem cell-based cell types for inherited and non-inherited macular degenerations. Study type: Interventional. Last updated 25 October 2016.

Sponsor	Cell Type or Intervention	Condition	Phase of Trial	Type of Delivery (Intervention)	ClinicalTrials.gov Identifier	Status
StemCells, Inc.	Human central nervous system stem cells (HuCNS-SC)	Geographic atrophy (GA) of age-related macular degeneration (AMD)	Phase I and II	Subretinal transplantation	NCT01632527	Completed
University of São Paulo	Autologous bone marrow stem cells	Macular degeneration	Phase I and II	Intravitreal injection	NCT01518127	Recruiting
Al-Azhar University	Autologous bone marrow stem cells	Dry, age-related macular degeneration (AMD)	Phase I and II	Intravitreal injection	NCT02016508	Unknown
Bioheart, Inc.	Adipose-derived stem cells	Dry, macular degeneration	Not reported	Intravitreal injection	NCT02024269	Withdrawn
Retina Association of South Florida	Bone-marrow delivered stem cells (BMSC)	Retinal disease	Not reported	Retrobulbar	NCT01920867	Recruiting
		Macular degeneration		Subtenon		
		Hereditary retinal dystrophy		Intravenous		
		Optic nerve disease		Intravitreal		
		Glaucoma		Intraocular		
University of California, Davis	CD34 + bone marrow stem cells	Non-exudative, age-related macular degeneration	Phase I	Intravitreal injection	NCT01736059	Enrolled by invitation
		Diabetic retinopathy				
		Retinal vein occlusion				
		Retinitis pigmentosa				
		Hereditary macular degeneration				
Red de Terapia Celular	Autologous bone marrow stem cells	Retinitis pigmentosa	Phase I	Intravitreal injection; subconjunctival injection of saline	NCT02280135	Recruiting
StemCells, Inc.	Human central nervous system stem cells (HuCNS-SC)	Age-related macular degeneration	Phase II	Subretinal transplantation	NCT02467634	Terminated; based on a business decision unrelated to any safety concerns

CD34, cell-cell adhesion factor that mediates the attachment of stem cells to bone marrow extracellular matrix.

**Table 3:** iPSC-derived RPE-based studies for inherited and non-inherited macular degenerations. Study type: Observational. Last updated 25 October 2016.

Sponsor	Cell Type	Condition	ClinicalTrials.gov Identifier	Status	Objective
Moorfields Eye Hospital	Human iPSC-derived RPE	Age-related macular degeneration	NCT02464956	Not yet recruiting	Successful production of a retinal epithelial layer of cells that fulfills Regulatory Regulation for Transplantation.
NHS Foundation Trust					
Mayo Clinic	Human iPSC-derived RPE	Autosomal recessive bestrophinopathy (ARB)	NCT02162953	Recruiting	To collect DNA, RNA, and skin samples from individuals with ARB or other diseases due to mutations in the gene BEST1. These models will be used to identify and test therapeutic approaches to treating these diseases.
		Best vitelliform macular dystrophy (BVMD)			
		Adult-onset vitelliform dystrophy (AVMD)			
		Autosomal dominant vitreoretinalchoroidopathy (ADVIRC)			
		Retinitis pigmentosa (RP)			
National Eye Institute (NEI)	Human iPSC-derived RPE		NCT01432847	Recruiting	To collect hair, skin, and blood samples to study three eye diseases that affect the retina (Best disease, L-ORD, and AMD)

iPSC, induced pluripotent stem cell; RPE, retinal pigment epithelium; NHS, National Health Service (of England); ARB, angiotensin receptor blockers; L-ORD, late-onset retinal degeneration; AMD, age-related macular degeneration.

## Abbreviations

iPSC	Induced pluripotent stem cell
AMD	Age-related macular degeneration
RPE	Retinal pigment epithelium
ESC	Embryonic stem cell
BM	Bruch’s membrane
ZO-1	Zonula occludens protein-1
GA	Geographic atrophy
HLA	Human leukocyte antigen
MHC	Major histocompatibility
FDA	U.S. Food and Drug Administration
ECM	Extracellular matrix
VEGF	Vascular endothelial growth factor
SOX2	(Sex determining region Y)-box 2
OCT3/4	Octamer-binding transcription factor 3/4
Klf4	Kruppel-like factor 4
c-MYC	Regulator gene that codes for a transcription factor; Myc
BEST1	RPE-specific protein bestrophin-1
